# Shockwave lithotripsy-assisted TAVI in a patient with severely calcified peripheral arteries and porcelain aorta

**DOI:** 10.1186/s43044-025-00645-z

**Published:** 2025-06-01

**Authors:** Uzeyir Rahimov, Rufat Zeynalov, Emin Karimli, Farid Aliyev, Elkhan Hajiyev, Khatira Abdulalimova, Shafag Mustafaeva, Teoman Kilic

**Affiliations:** 1 Universal Hospital, Baku, Azerbaijan; 2 Baku Health Center, Baku, Azerbaijan; 3 Kocaeli University, İzmit, Turkey

**Keywords:** TAVI, Aortic stenosis, Lithotripsy

## Abstract

**Background:**

Transcatheter aortic valve implantation (TAVI) is the standard treatment for severe aortic stenosis (AS), particularly in high-risk patients. However, peripheral artery disease with extensive vascular calcification poses challenges for transfemoral access. Shockwave intravascular lithotripsy (IVL) has emerged as a promising technique to facilitate vascular access in such cases.

**Case presentation:**

A 73-year-old male presented with non-ST elevation myocardial infarction. His condition necessitated urgent coronary revascularization and later, TAVI for severe AS. Pre-procedural computed tomography angiography revealed severe circumferential calcification of the entire aorta and both iliac and femoral arteries. Due to the extensive calcification and stenosis of the left common iliac artery, IVL was performed to optimize vessel diameter prior to TAVI. Post-IVL, optimal luminal expansion was achieved (6.8 mm), allowing safe passage of the delivery sheath.

**Conclusion:**

IVL-assisted transfemoral TAVI is a safe and effective strategy in patients with extensive iliofemoral calcifications.

**Supplementary Information:**

The online version contains supplementary material available at 10.1186/s43044-025-00645-z.

## Background

Transcatheter aortic valve implantation (TAVI) has become the standard treatment for severe aortic stenosis (AS), particularly in elderly patients with high surgical risk [1–3]. However, peripheral artery disease (PAD) remains a significant challenge for transfemoral access due to severe vascular calcifications [4]. Shockwave intravascular lithotripsy (IVL) has emerged as a promising technique to facilitate transfemoral TAVI in patients with extensive vascular calcification [5]. In this report, IVL-assisted transfemoral TAVI in a patient with severe circumferential peripheral arterial anatomy is presented.

## Case report

A 73-year-old male with a history of coronary artery disease (CAD), prior percutaneous coronary intervention (PCI), critical AS, end-stage renal disease on dialysis, and congestive heart failure presented with non-ST elevation myocardial infarction (NSTEMI). His condition necessitated urgent coronary revascularization and later TAVI for severe AS.

**Fig. 1:**
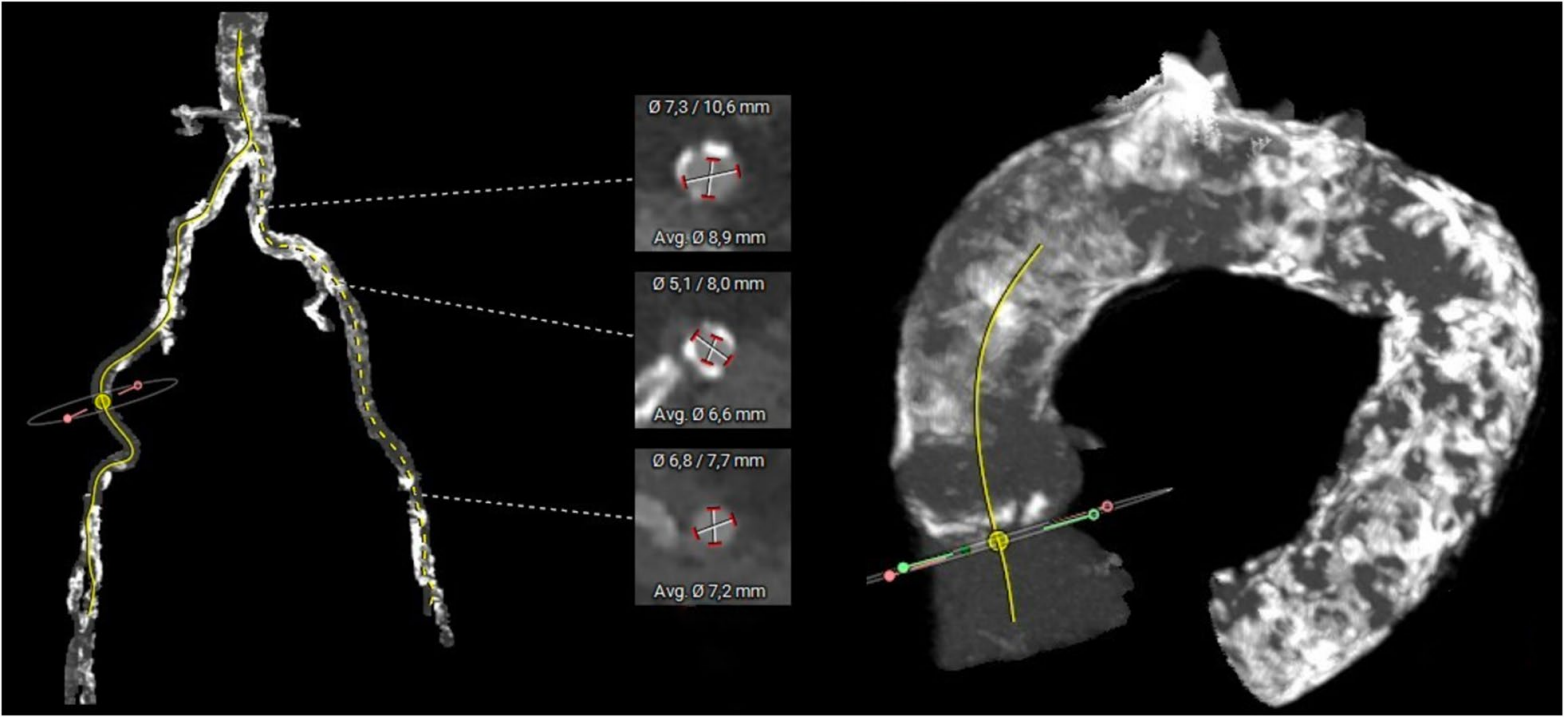
Left—Descending aorta and iliac arteries, Right—Porcelain aorta

Pre-procedural computed tomography (CT) angiography revealed severe circumferential calcification of the entire aorta and both iliac and femoral arteries. The left femoral artery was chosen for access due to a larger lumen diameter (5.1 mm) compared to the right (4.3 mm). Due to the extensive calcification and stenosis of the left common iliac artery, IVL was performed to optimize vessel diameter prior to TAVI (Fig. 1).

**Fig. 2:**
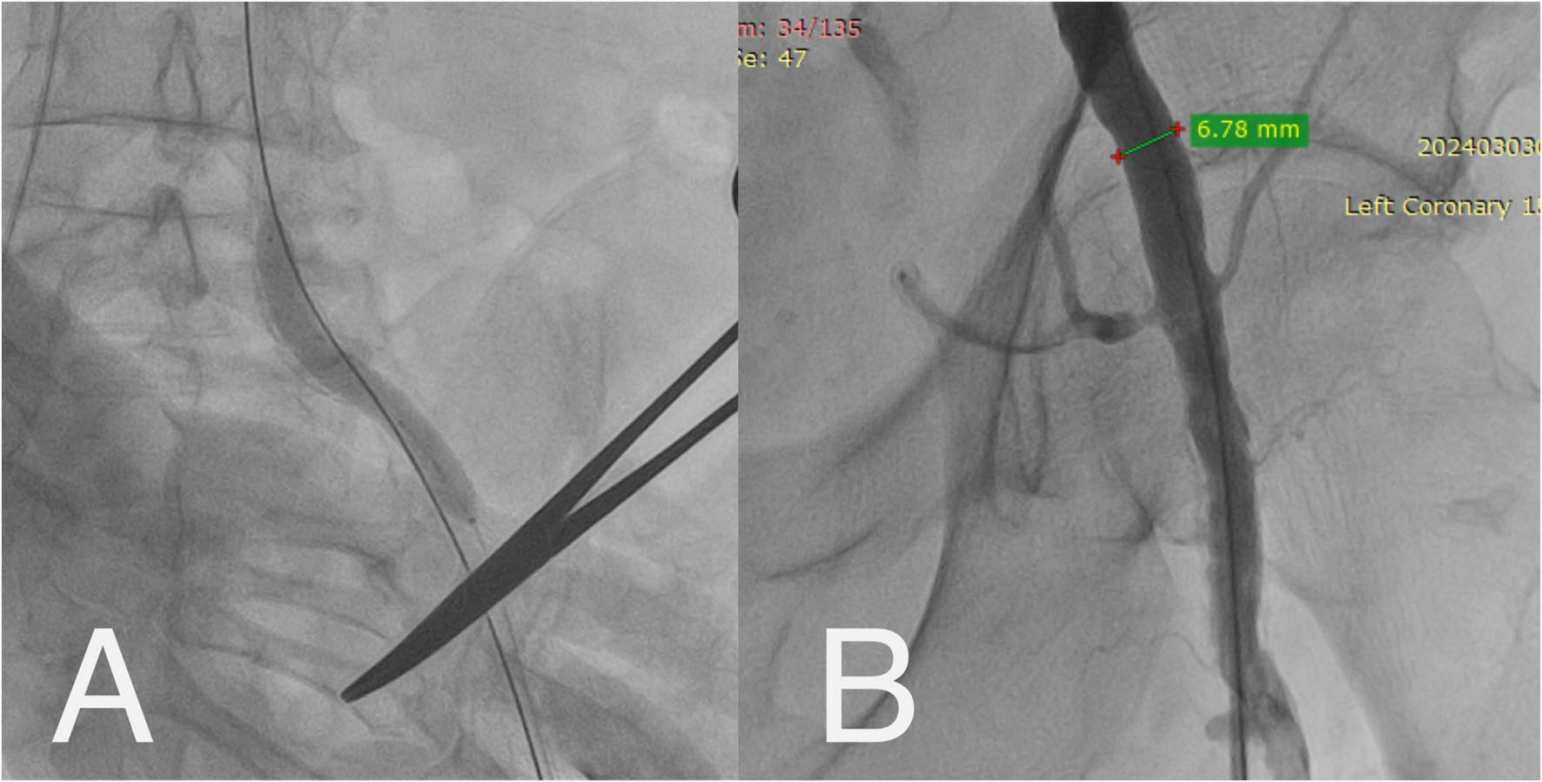
**A**—Intravascular lithotripsy, **B**—Diameter of left iliac artery post-intravascular lithotripsy

Following the deployment of a closure device, a 6 Fr sheath was inserted into the left femoral artery. IVL (Shockwave M5, 7 mm) was delivered over a 0.014-inch guidewire to the calcified iliac stenosis (Fig. 2A). Multiple pulses were applied at low pressure (4 atm.) to complete the full cycle of 300 pulses per balloon. Post-IVL, optimal luminal expansion was achieved (6.8 mm), allowing safe passage of the delivery sheath (Fig. 2B).

**Fig. 3:**
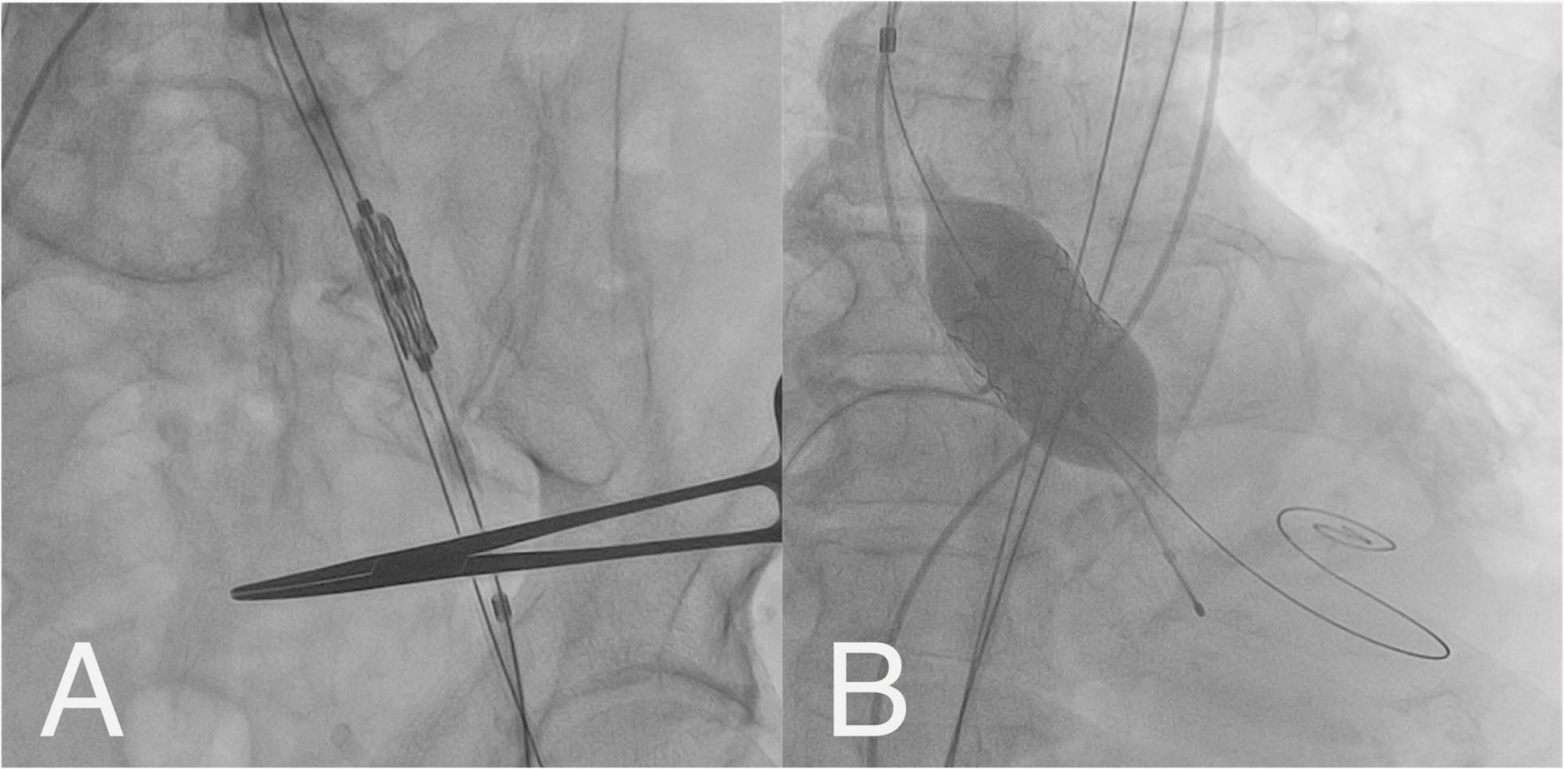
**A**—Advancement of prosthetic valve through iliac artery, **B**—Implantation of prosthetic valve

A 29 mm MyVal balloon-expandable valve was implanted via the transfemoral approach. The procedure was successful, with significant improvement in gradients (Pmean: 6 mmHg, Pmax: 13 mmHg) (Fig. 3). The femoral access site was closed using ProGlide. The patient remained hemodynamically stable with no neurological deficits post-procedure and was discharged in stable condition three days later.

## Discussion

Managing older patients with severe AS and coexisting PAD, CAD, CKD, and DM remains challenging, particularly when deciding between surgical and percutaneous approaches. This decision requires a careful balance of risks and benefits.

Severe PAD poses a challenge for transfemoral TAVI, often necessitating alternative access routes associated with higher complication rates. IVL has proven effective in modifying vascular calcifications, enabling transfemoral TAVI in high-risk patients. In our case, IVL facilitated safe arterial access, avoiding alternative approaches and their associated risks.

Porcelain aorta is present in 5.2% of TAVI patients and increases stroke risk. Studies indicate higher myocardial ischemia rates and procedural stroke risk in porcelain aorta patients, particularly with transapical access [6, 7]. In our case, severe aortic arch calcification increased stroke risk, supporting the transfemoral approach. In patients with extensive aortic calcifications, the use of a balloon-expandable valve minimized the risk of embolization. Furthermore, PCI prior to TAVI was crucial in optimizing myocardial perfusion, given the patient’s high ischemic risk. The French National TAVI Registry (FRANCE 2) found that significant LAD stenosis increases three-year mortality [8]. Current guidelines recommend PCI before TAVI in patients with severe CAD, especially those with ACS. Our patient presented with NSTEMI, leading us to perform revascularization first, though long-term outcomes remain uncertain.

IVL is a promising adjunct in TAVI, especially for patients with severely calcified peripheral arteries. This technique allows safe transfemoral access, reducing the need for higher-risk alternative approaches. Future studies should further evaluate IVL’s long-term outcomes in TAVI patients.

## Conclusion

IVL-assisted transfemoral TAVI is a safe and effective strategy in patients with extensive iliofemoral calcifications. It optimizes vascular access and reduces complications, making transfemoral TAVI feasible even in high-risk patients. The successful use of IVL in our case highlights its potential in complex vascular anatomies, supporting its broader adoption in structural heart interventions.

## Supplementary Information

Additional file 1.

## Data Availability

No datasets were generated or analyzed during the current study.

## Abbreviations

TAVI: Transcatheter aortic valve implantation
AS: Aortic stenosis
PAD: Peripheral artery disease
IVL: Intravascular lithotripsy
CAD: Coronary artery disease
PCI: Percutaneous coronary intervention
NSTEMI: Non-ST elevation myocardial infarction
CT: Computed tomography
